# 43. Auto-splenectomy and malignancy: complications unheard of in sarcoidosis

**DOI:** 10.1093/rap/rky034.006

**Published:** 2018-09-20

**Authors:** Premila Kadamban, Rasha Brier, Sundeept Bhalara

**Affiliations:** 1 Rheumatology, Basildon and Thurrock University Hospital, Basildon, UNITED KINGDOM; 2 Rheumatology, Watford general hospital, Watford, UNITED KINGDOM

**Introduction:** Sarcoidosis is a multi-system granulomatous inflammatory disease of unknown aetiology. Although many patients with sarcoidosis resolve spontaneously, a significant proportion of patients have chronic or progressive disease with resultant morbidity. Auto-splenectomy is a well-known complication of sickle cell disease; which is caused by micro vascular occlusion by deformed red blood cells which leads to ischaemia and infarction. Repeated infarctions lead to necrosis and gradual resorption of spleen. To our knowledge it was never reported to have occurred in any patients with sickle cell traits or systemic sarcoidosis. However splenic infarction was observed both in sickle cell traits and in patients with sarcoidosis in the past. Probably this is the first case of auto-splenectomy described either in sarcoidosis or in sickle cell traits. Chronic inflammation is associated with an increased risk for malignant lymphomas or cancer in the affected tissues. Theoretically this would apply also to sarcoidosis. Little is known about the cancer incidence following sarcoidosis. Case series have suggested an increased incidence of malignant lymphomas.

**Case description:** This 23 year old gentleman first presented at the age of 10 with intermittent headache, widespread arthralgia, painful skin rash on buttocks and shins and fatigue. He was known to have sickle cell trait. He was found to have bilateral pan-uveitis, erythema nodosum and lymphadenopathy. Further investigation revealed high inflammatory markers and elevated serum ACE. The skin biopsy demonstrated non-caseation granuloma. Based on these he was diagnosed with sarcoidosis and commenced on steroids followed by methotrexate. He had remarkable response to the treatment and remained clinically well during the following six years; therefore the treatment was weaned off and stopped altogether. The constitutional symptoms recurred after three months of stopping treatment and he started to lose weight. He was found to have palpable spleen. His CT scan of the chest abdomen and pelvis showed extensive intra-abdominal small volume lymphadenopathy and a large spleen (13 cm wide) with a couple of splenic granulomas. Blood results showed high inflammatory markers and elevated serum ACE. Lympho- proliferative disorder was considered as a differential diagnosis initially and was disregarded later as the lymph nodes were of small volume (<11mm) and splenic granulomatous lesions have not been described in lymphoma. A few weeks later. the patient developed erythema nodosum and a similar violaceous rash as before. Therefore he was diagnosed to have a relapse of sarcoidosis and he restarted treatment with steroids and methotrexate. He had good clinical response and a follow up scan revealed resolution of intra- abdominal lymphadenopathy. There was an incidental finding of parenchymal infiltrative changes in relation to right lung middle lobe. But the patient never had any respiratory symptoms. He remained clinically very stable in sarcoidosis point of view during the following six years. But he had couple of visits to the Accident and Emergency unit with a history of left sided chest pain; was thoroughly investigated including a CTPA and no cause was found. After three years into the development of relapse he had a follow up scan which showed resolution of splenic granulomas but the splenic parenchyma was heterogeneous in appearance with low density areas. Few years later patient had a relapse due to poor compliance and a repeat CT scan again showed extensive intra-abdominal lymphadenopathy, multiple hepatic and renal granulomas and pulmonary infiltrate with a nodule in the right lower lobe. The other important finding was that the spleen was not visualised which was clearly seen three years before. The patient did not undergo surgical splenectomy in between. Therefore he was declared to have had auto-splenectomy. The patient restarted steroids and methotrexate and had good response. Clinically he improved and the inflammatory markers normalised. In view of the persistent infiltrative and nodular changes in the right lower lobe the patient had a PET scan which confirmed the presence of a nodule in the right lower lobe with high uptake. As the nodule was not amenable to bronchoscopy guided biopsy patient had an open biopsy the histology of which showed muco-epidermoid tumour. Following this the patient had right lower lobectomy. Chemotherapy was not indicated as the tumour was low grade. Following the diagnosis of tumour methotrexate was switched to azathioprine. Currently his disease is well controlled on azathioprine and a small dose of prednisolone. In view of the absence of spleen he was advised to have regular prophylactic antibiotics and meningococcal,pPneumococcal and Hib vaccines. He was also advised to have malaria prophylaxis before visiting endemic regions.

**Discussion:** The process of auto-splenectomy starts very early in life in sickle cell disease. Usually there is evidence of hypo-splenism before 12 months of age in the majority of children. Repeated splenic vaso-occlusion leads to fibrosis and progressive atrophy of the organ (auto-splenectomy), which is generally complete by the age of 5 in sickle cell disease. Splenic involvement is not very common in sarcoidosis. Only 10% of the patients have quantifiable splenomegaly; while 3% of them have massive splenomegaly. Mostly the patients are asymptomatic. But massive splenic infarction was reported in few cases. Patients with sarcoidosis have been shown to have impaired vascular endothelial function and increased arterial stiffness. Conceivably, chronic perivascular inflammation resulting in the peri-arteriolar fibrosis seen on histology may have compromised vascular supply, leading to visceral ischaemia and infarction. Sickle cell trait occurs in approximately 300 million people worldwide. It’s long considered to be a benign condition. Splenic Infarction occurs in patients with sickle cell trait and it is more common with exercise at high altitude but has occurred with altitude exposure at rest or with exercise at sea level. In this particular case both pathologies could have contributed to auto-splenectomy. Retrospectively, this patient’s admission and A&E visit with left sided chest pain which didn’t have any explanations at that time could have been due to recurrent splenic infarctions. Endothelial dysfunction and chronic peri-vascular inflammation along with micro vascular occlusion could have caused multiple small infarctions which would have led to complete resorption of the spleen. Patients with sarcoidosis appear to be at increased risk of cancer particularly for cancers of the lung, stomach, small intestine, and liver, for melanoma and non-melanoma skin cancer, non-Hodgkin’s lymphoma, and leukaemia The estimated overall relative risk for cancer is equal to or slightly higher than that seen in other chronic diseases such as diabetes, inflammatory bowel disease, and rheumatoid arthritis. Recent data have expanded the concept that inflammation is a critical component of tumour progression. Many cancers arise from sites of infection, chronic irritation and inflammation. It is now becoming clear that the tumour microenvironment, which is largely orchestrated by inflammatory cells, is an indispensable participant in the neoplastic process. This patient had a sub-clinical lung involvement for a long time which was demonstrated as an infiltrative lesion in right lower lobe 6 years prior to the demonstration of a nodule in the same area. Chronic poorly controlled sarcoidosis has certainly contributed to the development of this malignant tumour in the lung in our patient who is a non-smoker.

**Key Learning Points:** Chronic inflammation leads to multiple detrimental complications including malignancy. It’s absolutely essential to control it with immune-modulatory therapy to minimise these complications. Splenic infarction and auto-splenectomy are possible complications of systemic sarcoidosis. Consider splenic infarction as the differential diagnosis if a sarcoidosis patient presents with chest pain or abdominal pain.

**Disclosure: P. Kadamban:** None. **R. Brier:** None. **S. Bhalara:** None.

